# Examination of Gender Differences: Causal Attributions of Treatment-Seeking Individuals With Overweight and Obesity

**DOI:** 10.32872/cpe.12089

**Published:** 2024-12-20

**Authors:** Carmen Henning, Caroline Seiferth, Tanja Färber, Magdalena Pape, Stephan Herpertz, Sabine Steins-Loeber, Jörg Wolstein

**Affiliations:** 1 Department of Psychopathology, University of Bamberg, Bamberg, Germany; 2 Department of Clinical Psychology and Psychotherapy, University of Bamberg, Bamberg, Germany; 3 Department of Psychosomatic Medicine and Psychotherapy, LWL-University Hospital, Ruhr University Bochum, Bochum, Germany; Friedrich-Alexander-Universität Erlangen-Nürnberg, Erlangen, Germany

**Keywords:** overweight, obesity, gender, causal attributions, physical activity, health behavior, mHealth

## Abstract

**Background:**

Addressing patients' perceptions of the causes of their overweight and obesity may be a promising approach to enhance treatment motivation and success. Previous research suggests that there are gender differences in these aspects. The objective of this study was to investigate gender differences in causal attributions among individuals with overweight and obesity who participated in a cognitive-behavioral mobile health (mHealth) intervention.

**Method:**

Causal attributions were assessed using the revised Illness Perceptions Questionnaire, which included a rated and open answering section. An ANCOVA was conducted for each causal factor (behavioral, psychological, risk, external) as a dependent variable to determine gender differences, which were analysed with chi-squared tests for open-ended responses.

**Results:**

The most frequently mentioned and highly rated cause was behavior for both genders (59.8% of 639 responses). The results indicated that women rated psychological causes, particularly stress-related causes, significantly higher, *F*(1,211) = 14.88, *p* < .001, η^2^ = .07, and were more likely to cite emotional eating than men, χ^2^(1, *N* = 639) = 15.06, *p* < .001. Men rated alcohol stronger as cause than women, *t*(125.05) = 3.79, *p* < .001.

**Conclusion:**

The findings of this study contribute to the understanding of the gender differences in causal attributions among individuals with overweight or obesity. Implementing stress management interventions with a focus on emotion regulation is pivotal, especially for females. Interventions should focus on sensitizing males to the association between emotions and eating behavior. The causal attributions should be assessed with different survey methods in order to match the patient’s view of their condition.

## Highlights

- Causal attributions of overweight and obesity differ between males and females.
- Female participants attributed their overweight/obesity primarily to psychological causes.
- Awareness of the association between emotion and overweight or obesity for males is necessary.
- For both genders, behavioral aspects and enhancing stress management should be focused on.

Nutrition and exercise programs for individuals with overweight or obesity (OO) are widely available, but the third pillar of evidence-based treatment, cognitive-behavioral interventions, is difficult to obtain at low-threshold. Health insurance companies cover the costs of mobile Health (mHealth) interventions in some countries (Roth et al., 2023), which can adequately bridge the long waiting times for specialized in-person treatment. Therefore, identifying the underlying mechanism of individuals with OO to engage with mHealth interventions that address cognitive-behavioral aspects of weight loss and weight-gain prevention is important.

The perception of causes among individuals with overweight (Body Mass Index, BMI = 25 – 29.99 kg/m^2^) or obesity (BMI ≥ 30 kg/m^2^) differs based on socio-cultural factors and self-perceived consequences. The media reinforces the ideal of thin women and the necessity of dieting for females (Pedersen, 2010), which can lead to an internalization of a thin beauty ideal and social comparisons (López-Guimerà et al., 2010). Women who have internalized the thin beauty ideal or show a high exposure to such media tend to report greater body dissatisfaction, unhealthy eating (López-Guimerà et al., 2010), and unrealistic weight goals (Dutton et al., 2010). In contrast, men seem to be less concerned about their OO and less aware of the consequences than females (Breland et al., 2023; Mozumdar & Liguori, 2011; Tronieri et al., 2017). Studies indicate that some males with OO do not perceive themselves as OO, whereas females with normal weight perceive themselves as overweight or obese (Chang & Christakis, 2003). Several gender differences in OO have been reported: overall, 53.5% of the German population is affected by overweight, including obesity, with a clear gender difference of 60.5% men and 46.6% women (Schienkiewitz et al., 2022). The prevalence of obesity is positively correlated with age and negatively correlated with socio-economic status (Schienkiewitz et al., 2022). Women are more likely to be affected by food craving (Hallam et al., 2016) and emotional eating behavior, i.e., overeating when experiencing (negative) emotions, than men, whereby this overeating reinforces negative emotions and can create a vicious circle (Breland et al., 2023). Research indicates that there is a higher prevalence of weight loss intentions among females than males (Houle-Johnson & Kakinami, 2018). Both genders are motivated to lose weight to improve overall health, but women also tend to report more internal motivators, such as increased personal esteem (Crane et al., 2017), while men tend to be more motivated by external factors, such as improved job performance (Sabinsky et al., 2007). In general, males are under-represented in obesity research, which often leads to difficulties in the transfer of research findings (Bramlage et al., 2004; Cooper et al., 2021; Pantalone et al., 2017).

Furthermore, research has demonstrated that women tend to associate obesity with more negative emotions and worse illness perceptions than men (Henning et al., 2022), and that this mental image is negatively associated with dieting attempts and weight cycling (Prill et al., 2021). Gender differences have also been found in the assumptions about the causes of an illness, the so-called causal attributions of one’s own OO. These causal attributions have direct effects on therapeutic outcomes, coping, and goal-related behavior (Mathieu et al., 2018; Zhang et al., 2018). These causal attributions can be categorized into different factors, e.g., psychological or genetic. The structure varies depending on the disease and its aetiology, whether it is multifactorial or can be attributed to a specific trigger (e.g., hereditary in the case of trisomy 21). For OO, which is a multifactorial disease, no unique structure has been identified (Daigle et al., 2019). Recent literature offers contradictory or non-comparable findings about the causal attributions of individuals with obesity, and most of the studies report no gender specific results. A cohort study with 75 individuals with OO suggested that unfavourable health behavior (e.g., excessive eating) was the most often causal attribution (58.7%) of own obesity, but individuals also considered psychological causes (e.g., worries) (Mathieu et al., 2018). Strong behavioral attributions (e.g., sedentary behavior) were also found in an investigation of individuals seeking surgical or behavioral/pharmacological weight loss treatment (Pearl et al., 2018). Agüera and colleagues (2021) categorized causal attributions, particularly for individuals with eating disorders, into four distinct categories: eating disorder*-*specific, psychological, risk, and external causes. The psychological factor included self-reported own behavior, but not eating behavior. This was categorized within the domain eating disorder specific causal factor, which makes it difficult to compare the results with other studies. Studies show that most of the individuals with OO named psychological causes, such as emotions, boredom, and low self-worth, followed by lifestyle aspects such as working environment (Agüera et al., 2021; Brogan & Hevey, 2009). Other causal attributions contained childhood experiences, social environment, medical reasons, eating behavior, and media influence. Brogan and Hevey (2009) conducted a network analysis, which showed that trauma, family problems, and an “addictive personality” were distal causes for overeating and comfort eating. Passive behavior, reduced physical activity levels, overeating, and comfort eating were proximal causes for obesity (Brogan & Hevey, 2009). To date, the majority of studies have not analysed results by gender. Consequently, the investigation of patterns of gender disparities with regard to causal attributions is underrepresented.

Several studies have examined the link between BMI and causal attributions of OO, but the results have been inconsistent. Lewis and colleagues (2010) suggested that the attribution of personal responsibility as a cause for obesity leads to powerlessness of the individuals with obesity grade III (BMI ≥ 40kg/m^2^) and to empowerment of individuals with lower BMI, whereas another study found an association with age but not the BMI level (Mathieu et al., 2018). Individuals with OO showed stronger attributions to heritability with their weight than normal weight individuals, which has been suggested to be associated with lower physical activity, decreased self-efficacy, and a low perception of personal control (Hilbert et al., 2009; Wang & Coups, 2010). However, their assumptions that their obesity was caused by overeating could have led to greater reported levels of physical activity (Wang & Coups, 2010).

The associations between causal attributions of OO and treatment outcomes or health behavior have been investigated by some studies. Individuals with OO showed more negative health outcomes as well as emotional and disinhibited eating behavior when they assumed psychosocial causes of their obesity (Mathieu et al., 2018). Psychosocial attributions were associated with pathologic eating patterns, which was more often prevalent in females (Mathieu et al., 2018). Research showed that interventions that match individuals’ causal assumptions of their illness can be a strategy to individualize treatment in OO and lead to better weight loss results (Bauer et al., 2020; Broadbent et al., 2009; Karekla et al., 2019).

Causal attributions are modifiable, disease and gender specific, and could lead to a change of health behavior (Bonsaksen et al., 2015; Surgenor et al., 2020; Zhang et al., 2018). A gender-sensitive investigation about causal attributions of individuals with OO, who are motivated to lose weight and interested in using mHealth for weight loss is lacking. The results could give an insight in underlying mechanisms and help enhance mHealth interventions for men and women. The aim of the present study was to examine these gender differences in this group. Given the contradictory or non-existent findings in the literature, we did not have directional hypotheses about gender-specific differences.

## Materials and Method

### Design

This cross-sectional study was part of the I-GENDO project, which was approved by the ethics committee of the University of Bamberg, Germany and the Institutional Review Board of the Ruhr-University Bochum (no. 18-6415) (Pape et al., 2022). The study was conducted in accordance with the Declaration of Helsinki. The participants provided their informed consent to participate in this study. Data collection took place via an online questionnaire between December 2019 and August 2020 within the pre-screening for the I-GENDO project. The aim of the project was the development and evaluation of a gender-sensitive mHealth intervention with psychological contents for weight loss and self-tailoring elements (Pape et al., 2022). After a telephone interview, individuals with suicidality or binge eating disorder were excluded. To avoid a systematic selection effect of a pseudo-random sample, we targeted especially males via press releases. Consequently, the sample is disproportionately stratified concerning gender, given that the proportion of males is still less than in the population.

### Sample

The study included 675 interested participants who were informed about the content of the project and screened for eligibility. The inclusion criteria of the participants were having overweight or obesity grade I and II (BMI = 25.00 – 39.9 kg/m^2^), being motivated to lose weight and interested in using an mHealth application, at least 18 years old, not pregnant, and having no binge eating disorder or bulimia nervosa according to DSM-5-criteria (American Psychiatric Association, 2020) (see Henning et al., 2024S, Additional File 1 for recruitment process). Individuals with a BMI greater than 40 kg/m^2^ often have comorbidities and drug therapy or bariatric surgery is advised (Deutsche Adipositas-Gesellschaft e.V., 2014). Consequently, they were excluded from the present study.

The final sample compromised 213 participants (female: 143; male: 70) between 19 and 71 years old. Power analyses were conducted using G*Power version 3.1.9.7 (Faul et al., 2007) and resulted in a sample size of 210 participants required to achieve 80% power for detecting a medium effect. More than half of the participants were married or in a partnership (55.4%), almost a third were single (31.4%) and 13.2% were divorced or widowed. Males and females did not differ in BMI (*Min:* 25.59kg/m^2^; *Max:* 39.88kg/m^2^), age or education (see Table 1).

**Table 1:** Sample Characteristics

Variable	Total (*n* = 213)	Females (*n* = 143)	Males (*n* = 70)	Group differences
**BMI** *M* (*SD*) (kg/m^2^)	33.35 (3.79)	33.51 (3.71)	33.01 (3.95)	*t*(211) = 0.301; *p* = .360
**Age** *M* (*SD*) (years)	46.45 (12.13)	44.94 (12.58)	49.51 (10.59)	*t*(160.05) = -2.78; *p* = .006
**Level of Education** (%)				χ^2^(2,213) = 2.14; *p* = .343
Low	13.62	13.29	14.28	
Middle	24.41	27.97	17.14	
High	59.62	57.34	64.29	

*Note.* Significance level *p* < .001.

### Instruments

We assessed the demographic variables such as age and gender at the beginning of the questionnaire. The causal attributions were assessed with the IPQ-R (Moss-Morris et al., 2002). First, 19 potential causes of OO (e.g., “stress or worries”) were presented and participants were asked to rate the extent of personal agreement with each cause on a 5-point Likert scale (1 = *strongly disagree*; 5 = *strongly agree*). Subsequently, participants were requested to name three causes that are most relevant to them personally in an open answering form.

#### Statistical Analysis

##### Rating of the Causes

**Figure 1:**
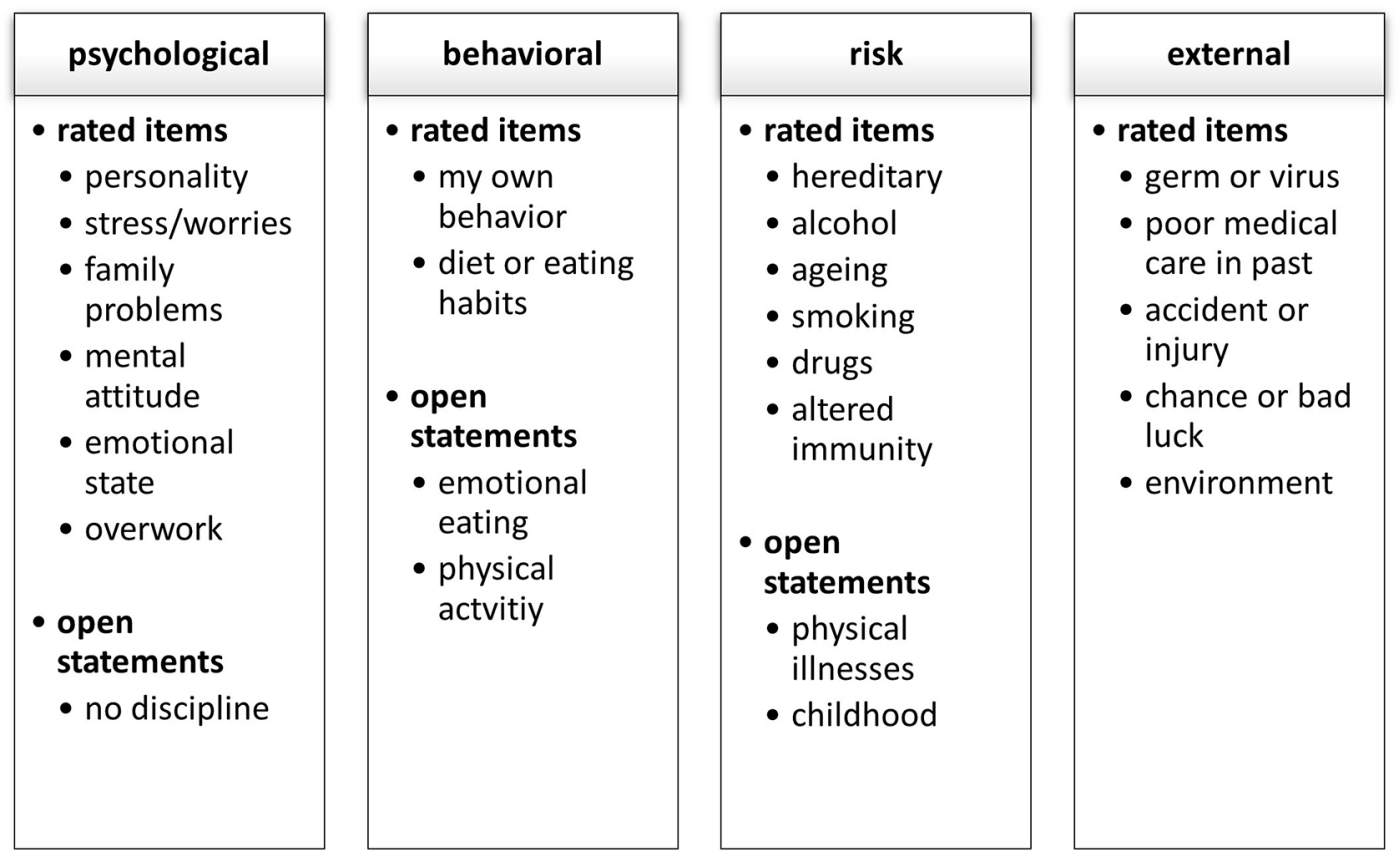
The 4-Factor Model of Causal Attributions

All analyses were conducted using IBM SPSS (Version 26). A four-factor model for causal attributions was set based on Moss-Morris and colleagues (2002), which was adapted for OO in accordance with the recommendations for this questionnaire (see Figure 1): psychological (6 items, Cronbach’s α = .746), behavioral (2 items, α = .750), risk (6 items, α = .413), and external (5 items, α = .646) factor. The significance level was set at *p* < .05 and was maintained through a Bonferroni correction for multiple testing (*p* < .001).

For each of the four causal factors a one-way ANCOVA was computed to analyse gender differences because the assumption of homogeneity of the regression slopes of gender for a MANCOVA was not met. We controlled for BMI and age in the first step and added gender as an independent variable in the second step. Additionally, we conducted two-sided *t*-tests for each item of the rated section.

The assumptions for ANCOVAs were checked: Homogeneity of regression slopes was not violated for three of the four dependent variables: behavioral, psychological, and risk factors (*p* < .0125). This assumption was not met for the external factor, as indicated by the significant interaction term for gender and age (*p* = .001). Consequently, we omitted age as a covariate in the ANCOVA for the external factor. The residuals were normally distributed for the psychological and risk factors as determined by the Shapiro-Wilk test (*p* > .05). However, the Shapiro-Wilk test was significant for the behavioral and external factors. The Kolmogorov Smirnov test was not significant (*p* > .0125) and because of the sample size, we omitted bootstrapping in the analysis. The assumptions of homogeneity of variances were not violated (Levene’s test: *ps* = .339 – .804). The leverage values (< .200) and values for Cook’s distance (< 1) indicated no outliers to be removed.

##### Open Statements

The open statements (*n* = 639) of the second questionnaire section were scalable, structured through deductive categorization according to Mayring (2015, p. 68) by two independent raters. The categorization was based on the 4-factor-model of the rated items with further additions (see Figure 1). For the 639 open statements, the degree of agreement by kappa was .946, which is an almost perfect interrater reliability (Landis & Koch, 1977). The analysis of the differences in frequencies for males and females were computed with chi-square tests. When expected cell frequencies were below five, we used the exact calculation option of SPSS.

## Results

A significant difference was found in the first part of the questionnaire for the psychological factor, with women rating the items as more likely to cause their obesity than men. In addition to psychological causes, men exhibited significantly stronger beliefs that alcohol was a possible cause. There was no significant gender difference at the factor level in the open response format. However, women were significantly more likely to report emotional eating as a cause of their obesity. Table 2 presents the descriptive statistics of the four causal attribution factors (psychological, behavioral, external, and risk) for the total sample and for females and males separately as well as the results of the ANCOVAs of the rated items section. All factors were weakly significantly correlated with each other (*r* = .177 – .228, *ps* < .001), but not with BMI or age.

**Table 2:** Descriptive Statistics of Causal Attributions and Results of ANCOVAs for Gender

Factor	Means (Standard Deviations)					*F*(1,211)	*p*	η^2^
	Total	Females	Males	Females^a^	Males^a^			
PSY	3.23 (0.86)	3.40 (0.80)	2.88 (0.88)	3.39 (0.07)	2.91 (0.10)	14.883	< .001	.066
BEH	4.54 (0.52)	4.56 (0.47)	4.50 (0.60)	4.56 (0.04)	4.51 (0.06)	0.440	.508	.002
RIS	2.33 (0.53)	2.33 (0.53)	2.35 (0.53)	2.34 (0.05)	2.33 (0.06)	0.002	.966	0
EXT	1.74 (0.55)	1.70 (0.54)	1.81 (0.57)	1.71 (0.05)	1.80 (0.07)	1.492	.223	.007

*Note.* PSY = psychological; BEH = behavioral; RIS = risk; EXT = external factor.

^a^Adjusted for BMI at all factors and for age in the psychological, behavioral, and risk factor.

### Psychological Causes

After controlling for age and BMI, a significant main effect of gender was found for the psychological causes. Females showed a stronger inclination towards psychological causes, with 'stress or worries' being the highest rated item on the scale (*M* = 4.15; *SD* = 0.80) compared to males (*M* = 3.71; *SD* = 0.95) (see Table 2). The approval rate for all items on the psychological scale (see Henning et al., 2024S, Additional File 2) was higher for females than for males. There was a significant effect of gender for the items 'stress/worries', *t*(118.14) = -3.34; *p* < .001, 'family problems', *t*(211) = 3.20; *p* < .001, and 'emotional state', *t*(211) = -4.79; *p* < .001 (see Henning et al., 2024S, Additional File 2).

Psychological causes were the second most frequently mentioned in the open-response format, with 25.4% for females and 23.8% for males. However, no significant gender difference was found (see Table 3). Although 'family problems' were rated significantly higher by females than males, men mentioned this cause more often than females in the open response section (refer to Henning et al., 2024S, Additional File 2 and Table 3).

**Table 3:** Frequencies, Percentages, and Results of the χ^2^(1; N = 639) Tests of the Open Statement Section

Factor / Subcategory	Frequency			Percentages			χ^2^	*p* ^a^	*V*
	Total	Females	Males	Total	Females	Males			
Emotional state	42	34	8	6.6	7.9	3.8	3.880	.049	.078
Family problems	9	3	6	1.4	0.7	2.9	4.728	.030	.086
Stress/worries	61	41	20	9.5	9.6	9.5	0	.989	.001
Work stress	11	7	4	1.7	1.6	1.9	0.062	.803	.010
No discipline	36	24	12	5.6	5.6	5.7	0.004	.951	.002
*Psychological total*	159	109	50	24.9	25.4	23.8	0.193	.661	.017
Diet or eating habits	212	131	81	33.2	30.5	38.6	4.106	.043	.080
Emotional eating	45	42	3	7.0	9.8	1.4	15.058	< .001***	.154
Physical activity	117	68	49	18.3	15.9	23.3	5.277	.022	.091
My own behavior	3	2	1	0.5	0.5	0.5	0	.986	.001
Habits (e.g., sleeping)	5	4	1	0.8	0.9	0.5	0.378	.539	.024
*Behavioral total*	382	247	135	59.8	57.6	64.3	2.640	.104	.064
Alcohol	16	5	11	2.5	1.2	5.2	9.579	.002	.122
(physical) Illness	43	37	6	6.7	8.6	2.9	7.472	.006	.108
Hereditary/past	13	10	3	2.0	2.3	1.4	.576	.448	.030
Pregnancy in past	5	5	0	0.8	1.2	0	2.457	.116	.062
*Risk total*	77	57	20	12.1	13.3	9.5	1.884	.170	.054
Environment	6	4	2	0.9	0.9	1.0	0.001	.980	.001
*External total*	6	4	2	0.9	0.9	1.0	0.001	.980	.001
*Others total*	15	12	3	2.3	2.8	1.4	1.152	.283	.042

*Note. V* = Cramer´s *V*, effect sizes of χ^2^ tests.

^a^Significance level due to Bonferroni correction *p* < .001 (***).

### Behavioral Causes

The participants rated the behavioral factor as the most important cause (see Table 2) and causes related to their behavior were most frequently mentioned in the open response section, both by females (57.6%) and males (64.3%) (see Table 3). Although the Chi-square test for the behavioral factor was nonsignificant, 'emotional eating' ('eating because I'm bored/frustrated') was reported as a cause of their OO significantly more often by women than men (see Table 3).

### Risk and External Causes

Neither the risk nor the external factor were in an area of agreement (see Table 2 for descriptive results and Henning et al., 2024S, Additional File 2 for single item agreement). Males rated 'alcohol' (*M* = 2.64; *SD* = 1.24) significantly higher than females, *M* = 1.98; *SD* = 1.12; *t*(125.05) = 3.79, *p* < .001 (see Henning et al., 2024S, Additional File 2) and reported it as a possible cause more often (see Table 3). Causes of risk were reported more frequently by females (13.3%) than males (9.5%), mainly due to physical illness (64.9% of female vs. 30.0% of male responses in this category), but again the difference was not significant (see Table 3). The number of statements categorized as external factors was less than 1% for both genders.

## Discussion

This study used an exploratory design to investigate the gender-related differences in self-perceived causes among individuals with OO. Participants were permitted to rate pre-defined causes and provide open-ended responses. All participants wanted to lose weight and participated in a project involving a behavioral-cognitive mHealth intervention. Responses were analysed at both the factor level (psychological, behavioral, external, and risk factors) and the item level.

In summary, significant gender differences were observed in the agreement with the psychological causes. Women considered stress, family problems and their emotional state to be significantly more important causes of their weight than men. Behavioral causes were rated most highly by both genders, with significantly more women than men citing emotional eating as a cause in the open-ended responses. The only cause for which gender differences were observed in both survey methods was alcohol consumption. This was rated significantly more strongly and cited more frequently by men.

The highest rated item on the psychological scale was 'stress/worries' for both genders, which emphasizes the importance of adaptive enhancing coping mechanisms in individuals with OO. Stress management training should be an integral part of psychological interventions, especially for females who showed significant higher scores on stress-related causal beliefs than males. Individuals with better coping strategies and competences to handle daily stresses are more successful in maintaining weight loss (Elfhag & Rössner, 2005).

The high rating of the importance of behavioral aspects such as eating and physical activity behavior is in line with results of other studies, which showed that they are proximal causes and causal attributions of obesity (Brogan & Hevey, 2009; Haslam & James, 2005; Mathieu et al., 2018; Pearl et al., 2018). Unfavourable health behavior such as emotional eating or physical inactivity seem to be maladaptive coping strategies for stress. Investigations of cardiac patients showed that individuals with beliefs in behavioral causes were more likely to change their dietary or exercise behavior (Weinman et al., 2000). Based on our results, which focused on a psychological mHealth weight-loss intervention, and existing research, it appears that motivating patients to address their OO could be effective by emphasizing the behavioral and changeable aspects of the condition.

This is supported by Fleary and Ettienne (2014) who found an association between the causal attribution of inactivity for males and their motivation to lose weight. Research has shown that males tend to benefit more than females from exercise in terms of weight loss and prefer this method instead of dieting and restrictive eating, which is perceived as a 'female approach' of weight management (Donnelly et al., 2003; Kiefer et al., 2005). Physical activity is not necessarily a prerequisite for weight-loss or maintenance because of compensatory behaviors and less discipline in attending sport programs regularly and on a long-term basis (Foright et al., 2018). Results about the effect of psychological intervention, such as behavioral change techniques on physical activity, are inconsistent (Awoke et al., 2022; Dombrowski et al., 2012). One possible approach could be to enhance self-efficacy by action planning, providing instruction and providing rewards to increase physical activity (Williams & French, 2011). The aim of psychological interventions in OO therapy could be to strengthen perseverance and reduce reward behavior related to food intake or alcohol consumption after exercise, particularly for men. The results indicate that men are aware of the role of diet and eating behavior in causing their overeating but are not aware of emotional eating (e.g., eating because of frustration). Interventions for males should focus on the association between emotions and overeating or alcohol consumption. Alcohol consumption seems to be a pivotal causal attribution of males, which is not surprising given that males drink more alcohol than females (Nolen-Hoeksema, 2004). It is recommended that men be made aware of the association between their own maladaptive coping, emotion regulation or self-rewarding behavior, which may manifest as alcohol consumption or eating, and their OO. Psychoeducational elements regarding the influence of alcohol on weight management in men should be considered in the development of mHealth interventions. In addition, self-monitoring of alcohol consumption may be useful for men, as this behavior change technique has been shown to be effective in interventions for physical activity and healthy eating (Samdal et al., 2017). Such a diary is easy to integrate into mHealth interventions but should be optional for the user or practitioner to activate, as alcohol consumption, especially in women, was not often reported as a suspected cause of OO.

It is suggested that emotional eating behavior for females should be focused on in interventions to target gender specific causal attributions. There is some evidence that females assume emotionally driven behavior such as emotional eating as a cause of their OO. Emotional eating has also been shown to be associated with the concept of food addiction (Pape et al., 2021) and eating addiction (Hebebrand & Gearhardt, 2021), to mediate the link between obesity, change in BMI and depression (Konttinen, Männistö, et al., 2010) and to be associated with less self-efficacy for the ability to maintain physical activity (Konttinen, Silventoinen, et al., 2010). Thus, emotional eating may represent a barrier to successful treatment. Our results are in accordance with other studies, which showed that females tend to engage in emotional eating behavior (Löffler et al., 2015). Our findings are also consistent with earlier observations, which showed that females had a much more 'emotional view' on their obesity and showed significantly stronger emotional illness representations than males (Henning et al., 2022). This means that women associate their OO with anger and guilt. Combined with the significant effect of gender at the second highest rated psychological factor, which was significantly more pronounced for women, the evidence emphasizes the importance of emotion-focused therapy in OO (mHealth) interventions, especially for females.

However, the results of our study with the two different survey methods also suggest that while men recognise the psychological component of their illness, they do not see it as being as strongly responsible for OO as women do. This can be seen from the fact that the gender differences disappear almost completely in the open responses. This phenomenon may be due to the fact that women are more aware of obesity and its consequences, e.g., health consequences, and suffer more from it than men (Audureau et al., 2016; Breland et al., 2023). This greater awareness could also lead them to participate more in weight management programmes. We therefore recommend considering this aspect in questionnaires for men that measure the strength of the perception of causes and consequences and, if necessary, that the survey be optimised by adding open questions or interviews. To encourage men to participate in weight loss interventions or research projects, it may be beneficial to reduce the emphasis on the perceived threat associated with such initiatives in recruitment activities. Instead, it may be more effective to focus on the elements of behavior that can be changed.

In contrast to other studies (Daigle et al., 2019), we did not find an association between causal attributions and BMI level. Our results are also not consistent with previous findings that individuals with a BMI of less than 40 kg/m^2^ believe in causes such as social aspects or environment (Daigle et al., 2019; Lewis et al., 2010). One potential explanation is that our treatment-seeking sample was motivated to lose weight. Consequently, they may have attributed their OO more often to changeable causes such as their behavior or coping mechanisms. The practical implication of this finding is that it is important to raise awareness of the impact of the environment, in order to enhance strategies to cope with these external stimuli. However, it is also important to emphasize their role, abilities, and potentials to meet these challenges, which in turn should enhance their self-efficacy to manage weight loss and maintenance.

Finally, a number of limitations need to be considered. The use of a cross-sectional design limits any causal conclusion. It is noteworthy that all respondents self-identified as either male or female, with no individuals selecting the "other" category. However, research in the domain of non-binary environments would be invaluable in order to facilitate the transfer of results to all individuals. Apart from these limitations, the generalizability of these results is limited because the sample consisted of individuals who were motivated to attend an mHealth study, which could have led to desirability effects in answering. The rated section excluded hedonistic items (e.g., eating because it tastes good), physical diseases, and physical activity, which might have led to a priming effect or bias in responding to the open-statement section. As with other studies, the reliability of the factors is low (Daigle et al., 2019), which is particularly evident in the "risk" factor, which in the context of OO encompasses a multitude of interrelated aspects. These include risky behaviors such as smoking, as well as external conditions, such as childhood experiences, which collectively contribute to a lack of internal consistency. We recommend an individualized view on a single item level respectively the subcategories of the open-statement section. The factorization seems to lead to a loss of information, which is needed for intervention planning. This might show the complexity and individuality of OO but could also be a chance for mHealth interventions as these can be individualized economically and easily.

The findings of this study contribute to the understanding of the gender differences in causal attributions among individuals with OO who are motivated to lose weight and interested in a psychological mHealth intervention. The practical implications are that implementing stress management interventions with a focus on emotion regulation is pivotal, especially for females. Interventions should focus on sensitizing males to the association between emotions and eating behavior. MHealth interventions that promote strategies to increase health behavior, such as physical activity and reducing alcohol consumption, may be more effective in engaging men than dieting or the proclamation of the consequences of OO. Furthermore, the causal attributions should be assessed with different survey methods in order to individualize (mHealth) interventions and to match the patient’s view of their overweight and target treatment options.

## Supplementary Materials

The Supplementary Materials contain the following items:

- The preregistration at ClinicalTrials.gov (NCT04080193) (Henning et al., 2019S-a)
- The preregistration at the German Clinical Trials Register (DRKS00016623) (Henning et al., 2019S-b)
- Additional information (Henning et al., 2024S):
  - Additional File 1: Recruitment process
  - Additional File 2: Single items analysis

HenningC.
SeiferthC.
FärberT.
PapeM.
HerpertzS.
Steins-LoeberS.
WolsteinJ.
 (2019S-a). Gender-sensitive enhancement of common weight loss strategies for overweight and obesity (I-GENDO)
[Preregistration at ClinicalTrials.gov; ID: NCT04080193]. PsychOpen. https://clinicaltrials.gov/study/NCT04080193


HenningC.
SeiferthC.
FärberT.
PapeM.
HerpertzS.
Steins-LoeberS.
WolsteinJ.
 (2019S-b). Gender-sensitive enhancement of common weight loss strategies for overweight and obesity: A personalized smartphone app
[Preregistration at the German Clinical Trials Register; ID: DRKS00016623]. PsychOpen. https://drks.de/search/en/trial/DRKS00016623


HenningC.
SeiferthC.
FärberT.
PapeM.
HerpertzS.
Steins-LoeberS.
WolsteinJ.
 (2024S). Supplementary materials to "Examination of gender differences: Causal attributions of treatment-seeking individuals with overweight and obesity"
[Additional information]. PsychOpen. 10.23668/psycharchives.15570
PMC1196056340177608

## Data Availability

The datasets and codes used or analysed during the current study are available from the corresponding author on request.

## Preregistration

ClinicalTrials.gov, NCT04080193, September 6, 2019; German Clinical Trials Register DRKS00016623, May 3, 2019
